# Reliability of Listener Judgments of Infant Vocal Imitation

**DOI:** 10.3389/fpsyg.2019.01340

**Published:** 2019-06-11

**Authors:** Helen L. Long, D. Kimbrough Oller, Dale A. Bowman

**Affiliations:** ^1^Infant Vocalizations Laboratory, School of Communication Sciences and Disorders, University of Memphis, Memphis, TN, United States; ^2^Konrad Lorenz Institute for Evolution and Cognition Research, Klosterneuburg, Austria; ^3^Institute for Intelligent Systems, University of Memphis, Memphis, TN, United States; ^4^Department of Mathematical Sciences HL, University of Memphis, Memphis, TN, United States

**Keywords:** infant vocalizations, infant imitation, prelinguistic vocal development, evolution of language, auditory perception, language development

## Abstract

There are many theories surrounding infant imitation; however, there is no research to our knowledge evaluating the reliability of listener perception of vocal imitation in prelinguistic infants. This paper evaluates intra- and inter-rater judgments on the degree of “imitativeness” in utterances of infants below 12 months of age. 18 listeners were presented audio segments selected from naturalistic recordings to represent in each case a parent vocal model followed by an infant utterance ranging from low to high degrees of imitativeness. The naturalistic data suggested vocal imitation occurred rarely across the first year, but strong intra- and inter-rater correlations were found for judgments of imitativeness. Our results suggest salience of the infant's vocal imitation despite its rare occurrence as well as active perception by listeners of the imitative signal. We discuss infant vocal imitation as a potential signal of well-being as perceived by caregivers.

## Introduction

Imitation has been widely studied in infant and child development (Meltzoff and Moore, 1977; Meltzoff, 1988a,b; Kugiumutzakis, 1999; Jones, 2007; Imafuku et al., 2019). Generally, the goal has been to seek insight about infant and children learning through imitation, with language learning being a special topic of interest (Bloom et al., 1974; Clark, 1977; Rodgon and Kurdek, 1977; Leonard et al., 1979; Moerk and Moerk, 1979). We have found no dispute in the child development literature regarding the importance of infant abilities to imitate as a foundation for language learning. But obvious instances of immediate imitation by infants of caregiver vocalizations do not occur very often (Pawlby, 1977; Papoušek and Papoušek, 1989; Užgiris et al., 1989). This raises the question of the possible importance of imitation by infants to parents in their understanding of the emergence of language in their children. To our knowledge no prior research has addressed the possible importance of parental awareness of vocal imitation by their infants.

We reason that in spite of the low rate of vocal imitation, caregivers are aware of infant *abilities* to imitate because imitation may constitute an important signal of the infant's learning and well-being whenever it does occur. Thus, we are studying the sense in which vocal imitation may be a fitness signal to caregivers. Specifically, we seek to better understand infant vocal imitation as a signal occurring in naturalistic interactions by using a continuous rating scale to assess adult listeners' perceptions of the imitativeness of infant vocalizations. By examining imitation in this way, we assess the reliability of infants' use of imitation as a vocal signal of their developmental status.

### Background

It is often claimed that babies learn language through imitation (Lewis, 1936; Mowrer, 1960; Bloom et al., 1974; Kugiumutzakis, 1999; Schreibman, 2005; Arbib et al., 2008; Ghazanfar, 2013). Others believe that infant imitation is present from birth as a way to map the actions of others who are “like me” onto a representation of their own actions to understand the psychological states of others and the self (Meltzoff, 2005, 2007) via active intermodal mapping (AIM) (Meltzoff and Moore, 1994, 1997) or via a mirror neuron system (Gallese and Goldman, 1998; Rizzolatti and Craighero, 2004; Simpson et al., 2015). These issues surrounding theories on the mechanisms and utility of infant imitation have been reviewed recently (Hurley and Chater, 2005a,b; Jones, 2009; Ray and Heyes, 2011; Oostenbroek et al., 2013; Keven and Akins, 2016). In this study, we do not seek to redefine or rediscover the mechanisms involved in the utility of infant imitation; rather, we seek to assess the salience of the infant's imitation as a signal for caregivers from an evolutionary developmental perspective.

Experimental studies make up the majority of research testing infants' capability to produce imitation, with the focus largely on imitation of facial gestures (Meltzoff and Moore, 1977, 1983; Meltzoff, 1988a; Heimann et al., 1989, 2017; Kuhl and Meltzoff, 1996). However, we know of no empirical evidence on the capacity for listeners to make consistent judgments about the degree of imitativeness of individual acts. The only data we know of on subjective judgments of infant imitation have been dichotomous ratings in experimental studies for the purposes of assessing coder reliability (Meltzoff and Moore, 1983, 1989; Meltzoff, 1988a,b; Barr et al., 1996; Carpenter et al., 1998; Collie and Hayne, 1999; Klein and Meltzoff, 1999; Sakkalou et al., 2013). This approach suggests that imitation is an all or nothing, binary skill. Our research will provide evidence of gradations in the extent of infant imitativeness and of the human listener ability to recognize such gradations.

Observational studies of infant vocal imitation have further provided an assessment of the frequency of imitation in parent-infant interactions (Masur, 2006). These, as well as experimental studies, require collecting subjective judgments on whether vocal acts are imitative (Užgiris, 2010). The occurrence of infant vocal imitation between ages 2 and 12 months in observational studies has been found to be low, occurring at <1 imitative event per min (Pawlby, 1977; Papoušek and Papoušek, 1989; Užgiris et al., 1989). It is important to note that these studies have identified instances of imitation using different criteria: Užgiris and Pawlby reported judgments of imitation on the basis of the *totality* of utterances, and “not on the basis of specific aspects such as pitch” (Užgiris and Pawlby, p. 111); in contrast, Papoušek and Papoušek evaluated imitative utterances by acoustic characteristics (i.e., pitch, duration, rhythm, and vowel or consonant resonance) and may have paid greater attention to the degrees in which utterances could be deemed imitative, thus potentially increasing the likelihood that utterances would be treated as imitative. However, these judgments remained binary. While dichotomous judgments of infant imitation may provide useful evidence on infant capability and frequency of occurrence, we find it necessary to assess the salience of imitation as a signal using listener judgments of degree of imitativeness.

### An Evolutionary Developmental Perspective on Infant Imitation

Within an evolutionary-developmental perspective (Bertossa, 2011; Oller et al., 2016), we propose that a key selection force on infant vocal imitation is based on the fact that it can be interpreted by parents as an indication of infant well-being, or fitness. Fitness is defined as the extent to which a biological trait is functional across a range of environments (Darwin, 1859; Latta, 2010). A reliable fitness signal used by infants would need to be salient and consistently perceived by listeners.

We follow the line of thinking that language emerges continuously with foundational capabilities building on each other (Oller, 2000; Oller et al., 2013). Specifically, early developmental skills and behaviors such as spontaneous vocalizations in the first month of life are seen within our perspective as foundational in building more complex skills such as canonical babbling and the infant's first words. The ability to imitate is also clearly foundational because learning to produce words requires being able to store and replicate phonological information. Thus, we seek to treat imitation as a feature of the emergence of language, recognizing that infant utterances can manifest varying degrees of imitation which can be interpreted by the caregiver as indicators of infant status in language learning.

Given that infant vocal imitation is infrequent, it would seem that parents must be acute in their identification of imitative utterances in order to make use of the information at all. In our longitudinal research we have noticed that parents in interviews with staff sometimes indicate sounds their infant can imitate, but we have not yet quantified these tendencies. Attentiveness to rarely occurring imitation events could suggest that parental attention to imitation ability was selected for through hominin evolution as an indicator of infant growth of the language capacity. This likelihood suggests it is important to empirically evaluate how reliably imitativeness is transmitted by the baby to potential caregivers. The potential importance of such work is also supported by widespread suggestions that the ability of infants to imitate is associated with positive language and cognitive development (Ramer, 1976; Réger, 1986; Snow, 1989; Masur and Eichorst, 2002; Sundqvist et al., 2016).

In spite of the existence of numerous studies of vocal imitation and its importance in predicting language development in infancy, there has never been any prior study of infant vocal imitation to our knowledge that has attempted to establish a “gold standard” for judgment of infant vocal imitation. Nor has any research to our knowledge addressed what acoustic properties of matching between parent-modeled and infant-responsive utterances would influence degrees of perceived imitation. Yet it seems undeniable that human adults *can* make judgments about infant and child vocal imitation—the key empirical questions are (1) to what extent would listeners agree with each other if they did make judgments of imitativeness when presented with paired parent-infant vocalizations, and (2) to what extent would they be consistent in their own judgments if they made them repeatedly?

To provide empirical answers to these questions is the primary goal of this paper. We consider such work to be prerequisite to establishing standards of judgment about the nature of infant vocal imitation and a requirement for the development of ultimate gold standards for other research involving observational judgments of imitation. We take an evolutionary perspective wherein it is assumed that human caregivers and potential human caregivers *must* be able to judge the vocalizations of human infants in terms of such issues as their speech-like quality, the degree to which they express distress, and the degree to which they conform to utterances produced by caregivers themselves (that is, the degree to which they are imitative). These abilities of caregivers, in accord with this evolutionary perspective, must be naturally selected because caregivers without such capabilities would be at a disadvantage in rearing successful children to compete for survival and reproduction. Thus, it seems that any normal human adult must be able to judge infant vocal imitativeness to some degree. We started our empirical work for this paper with the assumption that such a capability would likely be present in any listener-participant with normal intellect.

How could we empirically evaluate such a capability? An obvious method is testing for inter- and intra-rater agreement on a substantial number of utterance pairs selected on an intuitive basis as showing a wide range of infant imitativeness. We reasoned that if any individual rater's judgments failed to show significant correlation with the ratings of a group of other persons, that individual would have been revealed as incapable of (or extremely poor in) judging imitation. The magnitude of observed correlations among raters would be reflective of the extent to which natural selection had yielded a strong signal of imitativeness in infant vocalizations as well as a strong capability in listeners to recognize that signal.

The evolutionary perspective also suggests that although we do not know what magnitude of agreement to expect among and within listeners, we can expect statistically significant agreement. As argued above, a human who is unable to recognize vocal imitation would be at a disadvantage in recognizing all aspects of speech signals, indeed would not likely be able to understand speech, nor to judge the content of vocalizations of babies. Such a person would be at a disadvantage in trying to make sense of the vocal communications of their own progeny, and the progeny would presumably experience negative selection pressure due to concomitant insensitive parenting. We reason thus, that after many generations of selection, all persons without any significant capability to judge imitation would have been weeded out.

While it is expected that all raters will be significantly able to judge imitativeness (i.e., would show significant agreement with other raters), the evolutionary perspective also predicts that there must be variation both within and among raters—all traits that are subject to natural selection must show variation (Darwin, 1859; West-Eberhard, 2003; Locke, 2009). Evolutionary theory therefore suggests we should attend to variation both within and across observers.

A key point about such research is that there is, at present, no basis for asserting a “gold standard” for judgment of imitative and non-imitative events. Although we assume all normal humans should be able to significantly judge imitation, how would we know that one person is better at it than another? Even following significant experience in working with and making judgments on imitation, there would be no empirical way to assess that a person is particularly good at judging imitation in the absence of a measure of that person's agreement with the standard of humanity in general on judgments of imitation. Thus, we presume that research determining agreement within and across a panel of normal human listeners is a prerequisite to the establishment of an empirical gold standard for judgments of imitation and for providing empirical perspective on the role of imitation as a salient fitness signal to caregivers.

## Methods

### Data Collection

Approval for the longitudinal research that produced data for this study was obtained from the University of Memphis Institutional Review Board for the Protection of Human Subjects. Data were acquired from archives of the longitudinal investigations on typically developing infants in and around Memphis, Tennessee, and all parents spoke English in the selected laboratory recordings. Recruitment for this archival data was conducted in child-birth education classes and by word of mouth. Parents or prospective parents of newborn infants were presented with a detailed consent form after having been interviewed as possible participants in the longitudinal recordings. One infant was exposed to Ukrainian and English at home, but all other infants were exposed to only English at home. Criteria for inclusion of infant participants included a lack of impairments of hearing, vision, language, or other developmental disorders.

We drew from archived audiovisual recordings of six parent-infant dyads (3 male, 3 female infants) representing naturalistic interactions in a laboratory setting. During recordings, the parent-infant pairs occupied a studio designed as a child play room with toys and books. Laboratory staff operated four or eight pan-tilt video cameras located in the corners of a recording room from an adjacent control room—there were three such recording laboratories at varying stages of the research. In all the laboratories, two channels of video were selected at each moment in time with the goal of recording (1) a full view of the interaction and (2) a close view of the infant's face. Both the parent and the infant wore high fidelity wireless microphones, with the infant microphone <10 cm from the infant's mouth. Detailed descriptive information regarding laboratory equipment used can be found in previous studies completed from this laboratory (Buder et al., 2008; Warlaumont et al., 2010).

Two laboratory recording sessions were selected from all 6 infants at approximately 3, 6, and 10 months, for a total of 36 recordings used to select utterances. The average length of sessions used for this study was 19 min (range: 12–22 min). These sessions were selected from longer recordings which often lasted around 60 min, during which parents were asked to interact with their infant or with a laboratory staff member. Demographics and recording age for each infant at each session are tabulated in Appendix A (Supplementary Material, Data Sheet 1).

### Identifying Functions of Infant Vocalizations

All infant vocalizations across the recordings were initially labeled in terms of illocutionary force, defined as potentially communicative functions of the utterances (Austin, 1962; Searle, 1969; Oller et al., 2016). We sought all possible instances of imitation, which was one of the illocutionary forces coded, in both interactive or non-interactive contexts throughout the recordings we examined. The coding was done within the Action Analysis Coding and Training software (AACT, Delgado et al., 2010), used and discussed in previous research from this laboratory (Warlaumont et al., 2010; Jhang et al., 2017; Yoo et al., 2018). Pre-linguistic infants express varying emotional content (i.e., positive, neutral, and negative) in early vocalizations beginning at birth (Oller et al., 2013; Jhang and Oller, 2017). Infants have been shown to have the capacity to produce a single vocal type with multiple illocutionary forces on different occasions, suggesting they possess the foundations necessary for the variable illocutions seen for words and sentences in mature language. Following this thinking, pre-linguistic infant vocalizations can be used during dyadic interaction with varying communicative intentions, or vocalizations can be internally driven and produced for the infant's own purposes. Viewed in this context, vocal imitation is a kind of illocution, a function performed when an infant produces a sound that reveals matching to a heard sound.

Vocalizations exhibiting any degree of imitation as judged by an adult listener (author 1) were selected as stimuli, taking into account auditory and acoustic characteristics such as matching pitch contour, number of syllables, and/or syllable types in both dyadic and non-dyadic contexts. A non-dyadic circumstance could be, for example, if an infant imitated a caregiver who was not talking to the infant but offering examples of infant utterances to a laboratory interviewer. A total of 6,474 utterances were labeled for illocutionary force (with one possible force being imitation) in the 36 recordings used in this study.

### Extraction of Stimulus Pairs

Our goal in stimulus selection was to acquire a set of infant vocalizations that represented the broad continuum from high imitativeness to no imitativeness from the 6,474 utterances. We do not assume that there exists a gold standard for categorizing infant utterances into three groups of high, low and no imitativeness, but we aimed to select utterances roughly equally in these three intuitively determined groups in order to ensure that we would have stimuli across the entire continuum. The groups were used as a heuristic for the selection process and were not theoretically important, so we did not endeavor to make the selections precisely equal in the three groups.

Only 299 infant utterances were identified as showing *any* degree of imitativeness, <5% of all the utterances in the recordings^1^. From these, 108 utterances along with the preceding parent utterances, were selected to be extracted and used as stimulus items for listener judgments. Sixty of these were designated intuitively as showing “low” imitativeness and 48 as showing “high” imitativeness. The remainder of the 299 imitative utterances were eliminated because they (1) had a low signal-to-noise ratio, (2) poor recording quality, (3) high parent-infant voice overlap, (4) repeated imitations (without repeated preceding adult models), or (5) speech occurring between the model and the imitation. Fifty-eight additional pairs were identified from the original 6,474 in the recordings as clearly not imitative and were extracted for the purposes of including non-imitative infant utterances in the stimulus set, as long as these utterance pairs were not disqualified by any of the 5 elimination criteria above. This procedure ensured a wide range of possible judgments on degree of imitation. A total of 166 stimulus pairs were therefore used for listener judgments. Appendix B in Supplementary Material provides a visualization of the flow for the selection of stimulus pairs and additional commentary. Also, in Supplementary Material we provide 10 example stimulus wave files used in this experiment. Appendix D includes means and standard deviations for the 10 wave files provided as exemplars rated across the 0-100 scale.

### Listeners and Rating Scale

Eighteen listeners were asked to rate the degree of imitation for each of the 166 pairs. The participants included 15 graduate assistants (MA, AuD, and PhD graduate students in the School of Communication Sciences and Disorders) and 3 staff members of the Infant Vocalizations (IVOC) Laboratory, all of whom were female. The listeners had no previous experience rating infant utterances on a continuous scale or making judgments of degree of imitativeness; however, all listeners had experience listening to infant sounds and identifying vocalization types (e.g., squeals, growls, vowel-like sounds, etc.) and canonical syllables^2^. The first author, who selected the stimulus pairs, also participated as a listener (hereafter, “Rater 1”).

### Rating Scale

A continuous rating scale (range 0–100) was presented to listeners in the AACT software environment (Delgado et al., 2010) for making judgments on the degree of imitativeness of infant utterances as compared to adult models. See Picture C1 in Appendix C, which provides a screen shot of the scale tool. The listeners, prior to hearing any of the stimuli, were shown a screen shot of the rating tool and it was explained to them that when using the tool they would merely click with a mouse pointer on any location within the scale each time they would hear a stimulus, and AACT would assign a number from 0 to 100 indicating the degree of imitativeness specified. Listeners were encouraged to use the entire scale^3^. The scaling tool was very easy to use, and none of the raters expressed any difficulty in managing the rating task.

### Instructions for Listeners

Listeners were presented minimal instructions on how to make judgments of infant imitativeness. Specifically, they were told to broadly consider auditory-acoustic characteristics such as duration, pitch, syllabicity, and articulation of the parent and infant utterances when making their judgments. Our goal was to encourage listeners to use their natural intuitions about infant vocalization and thus hopefully for them to simulate mothers' judgments of imitativeness. Because these pairs were selected from infants younger than 12 months of age, listeners were encouraged to rate the degree of imitation regardless of whether the infant utterance was exactly like that of the caregiver (e.g., a word imitation).

### Calibration Stimulus Pairs

In order to ensure listeners understood the task, 12 calibration pairs were selected from the 166 stimuli by the first author and presented prior to the judgment task as examples of very high (6 pairs) or very low (6 pairs) degrees of imitativeness within the sample. The calibration pairs were not rated by the listeners during this presentation. These pairs were also included and randomized in order within the stimulus set in the full listening judgment task. Mean ratings for the calibration items that were made by the listeners during the full judgment task, along with ratings for the other stimulus pairs, can be seen in the discussion on rating scale usage in Appendix C2 of Supplementary Material.

### Listening Judgment Task (Rating Trials)

After listeners were presented instructions and the calibration stimulus pairs, the formal rating task began, with five randomized trial blocks of the 166 pairs presented to each listener. In other words, all 166 pairs (including the 12 calibration pairs) were presented five times to each listener for a total of 830 rating trials. The set of 166 stimulus pairs was randomized within each trial block. The beginning and ends of each trial block were inspected to ensure no single pair was presented twice within 10 consecutive stimulus pairs. The task took approximately one and a half hours for each listener to complete.

## Results

To ensure that the scale was being utilized appropriately, we first examined the range of ratings used by all the listeners. Zeroes occurred commonly in the ratings, and the highest minimum rating by any individual was 2 (mean minimum rating across all listeners: 0.3); ratings of 100 were also fairly common, and the lowest maximum rating by any individual listener was 97 (mean maximum: 99.4). Almost 2/3 of the ratings occurred in the middle of the scale from 20 to 75. All listeners were thus confirmed to have utilized essentially the entire scale for their judgments. See Appendix C for graphic analyses of rating scale usage and mean rater bias.

### Inter-Rater Correlations

**Figure 1:**
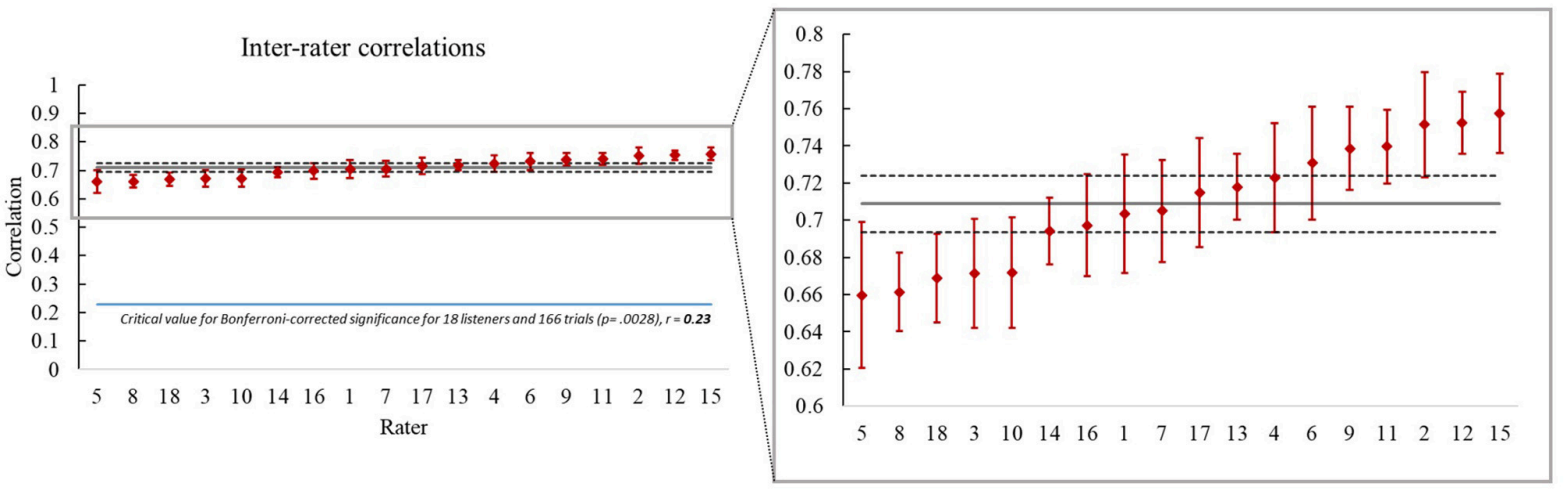
Mean inter-rater correlations (MICs) ordered from lowest (*r* = 0.66) to highest (*r* = 0.76) for each of the 18 raters represented by red diamonds. On the left, the entire correlational scale is represented, and on the right a blowup is offered for the region where the scores occurred. On both the left and right, the solid horizontal gray line represents the mean (0.71) across the 18 MICs and the dotted gray lines correspond to the 95% confidence interval *(0.694–0.724)* for that mean. On the left we also show, with a horizontal *blue* line, the critical value for statistical significance of the correlations; the huge gap between the critical value correlation and the actual correlations makes clear that the ratings of all the listeners were correlated at a highly significant level (*p* < 0.00001) with those of the other raters. At the same time the blowup on the right makes it possible to easily examine differences among the 18 listeners in their levels of agreement with the other listeners by evaluating the means and 95% CIs (the error bars) for any pair of listeners. For example, Rater 5 agreed significantly less with the others than Rater 15, since their CIs do not overlap at all. To compare any two raters' levels of agreement with that of any of the other raters, observe the error bars of one with respect to the mean of the other; if the CIs for the first rater do not overlap with the mean for the other rater, the two are significantly different at *p* < 0.05.

To compute mean inter-rater correlations (MICs), we first calculated the mean rating across the 5 trials on each stimulus pair for each listener. We will refer to these as the individual rater means (IRMs). We paired the IRM for each stimulus and for each rater with the IRMs of all the other raters and computed the 17 correlations for the pairings (*n* = 166). An MIC was calculated for each rater across these 17 pairings, and each of these MICs is represented in Figure 1 as a red diamond. The mean of the MICs, 0.71 (range: 0.66 to 0.76, *n* = 166 for each), was highly significant, *p* < 0.00001 (SD across the 18 MICs = 0.03, 95% CI [0.72, 0.69]). Even the lowest of these inter-rater correlations was highly significant (*p* < 0.00001, *n* = 166). These mean inter-rater correlations suggest moderate to strong positive relationships across raters for judgments on each stimulus pair, as expected based on the assumption that all normal human listeners should have an evolved capacity for recognizing vocal imitation. Although the agreement among listeners was highly significant, it is also true that the listeners showed notable and often significant differences from each other in the degree to which they agreed with the other listeners, as indicated by the error bars (95% CIs) of the MICs in Figure 1.

### Intra-Rater Correlations

**Figure 2:**
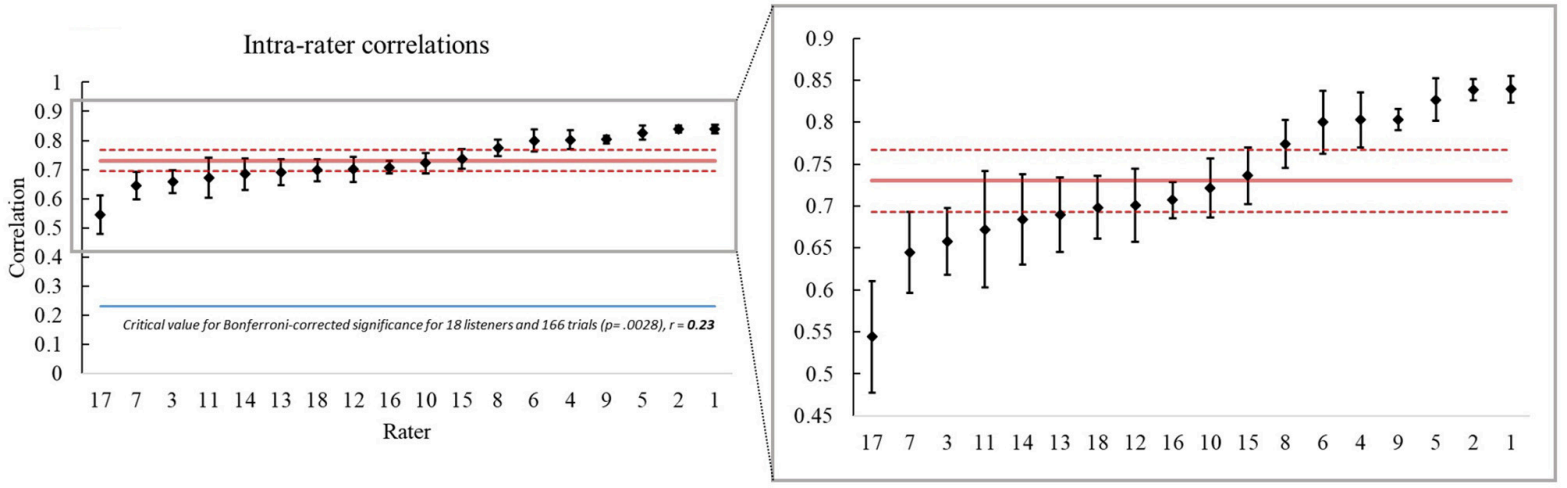
Mean intra-rater correlations organized from lowest (*r* = 0.54) to highest (*r* = 0.84) for all 18 listeners, with an average correlation of 0.73 indicated by the solid red line with a 95% CI of 0.69 −0.77 represented by dotted red lines. Intra-rater correlations were calculated for each of the 18 listeners by averaging the correlations of their ratings across all possible pairings of the five trial blocks for each of the listeners. The left-right distinction is as in Figure 1. Again, on the left there is a huge gap between the critical value correlation (blue line) and the actual correlations across the 18 listeners, making clear that all the intra-rater correlations were highly significant (*p* < 0.00001). Again, the blowup on the right makes it possible to easily examine differences among the 18 listeners in their levels of agreement with their own ratings across the 5 trial blocks, i.e., their rating consistency.

Intra-rater correlations, which reflect listener consistency of rating across trial blocks, were calculated from the mean within-rater correlations of the 166 IRMs across each of the 5 trials on each stimulus; all 10 possible pairings of the five trials for each listener were correlated. The average intra-rater correlation was *r* = 0.73 (*p* < 0.00001 for all ratings, SD = 0.08, 95% CI = 0.69, 0.77) ranging from 0.54 to 0.84. These results suggest moderate to strong positive relationships between individual rater judgments of each stimulus pair across trial blocks as shown in Figure 2, with the mean within-rater correlation for each listener represented as a black diamond.

As with inter-rater agreement, the very significant intra-rater agreement was also accompanied by differences among the raters in the degree to which they showed consistency in rating across the five trials. These differences are again reflected in means and CIs for the individual listeners in the figure.

### Intra-Rater Bias: Change in Ratings Over Trials

We also evaluated the statistical significance of the within-rater differences in rating levels between individual trials. Unlike the intra-rater correlation, this analysis compares each listeners' rating levels, comparing those levels for each trial block with all the other trial blocks (again in all 10 possible pairings), providing information on how raters changed their rating biases over time (e.g., higher or lower average ratings trial to trial).

Column 1 in Table 1 indicates the rater, and the subsequent columns indicate the *p-*values for the 10 possible pairings across 5 trials for each stimulus pair. A Kolmogorov-Smirnov test was conducted for each pairing. This is a non-parametric test of distributions, which can detect differences in mean ratings across trials or differences in distribution shape across trials. The null hypothesis for this test was that the ratings from the two trials came from the same distribution. In other words, a *p* > 0.05 indicates the two paired trials were not significantly different from each other. For example, the ratings from the first two trials for Rater 1 were not significantly different from each other (*p* = 0.349). The first and third, on the other hand, were in fact significantly different (*p* = 0.006). The third, fourth, and fifth ratings were not statistically different — *p* = 0.779, 0.689, 0.507, respectively. Three of the 10 pairings for Rater 1 showed statistically significant differences of ratings.

**Table 1:** Kolmogorov-Smirnov test for within-rater pairings across the 5 trial blocks (10 possible pairings).

	***p*****-value of test of agreement across judgments within rater**									
**Rater**	**1–2**	**1–3**	**1–4**	**1–5**	**2–3**	**2–4**	**2–5**	**3–4**	**3–5**	**4–5**
1	0.349	0.006	0.013	0.009	0.108	0.108	0.108	0.779	0.689	0.507
2	0.779	0.283	0.424	0.083	0.859	0.991	0.180	0.968	0.018	0.14
3	0.108	<0.001	0.002	0.034	0.062	0.349	0.689	0.083	0.002	0.507
4	0.924	0.424	0.227	0.018	0.779	0.507	0.062	0.998	0.507	0.507
5	0.283	0.140	0.001	0.083	0.083	<0.001	<0.001	0.507	0.108	0.689
6	0.227	0.140	0.062	0.108	0.991	0.859	0.689	0.689	0.968	0.349
7	0.013	0.006	0.507	0.596	0.779	0.349	0.083	0.108	0.013	0.689
8	0.025	0.227	0.083	0.083	0.968	0.046	0.034	0.349	0.283	0.779
9	0.507	0.424	0.004	0.004	0.006	<0.001	<0.001	0.424	0.424	0.924
10	0.596	0.068	0.689	0.013	0.09	0.968	0.018	0.284	0.848	0.227
11	0.859	0.006	<0.001	<0.001	0.034	<0.001	<0.001	0.006	0.001	0.859
12	0.859	0.227	0.924	0.083	0.596	0.998	0.227	0.349	0.779	0.227
13	0.018	0.006	0.025	0.227	0.001	0.227	0.025	0.046	0.001	0.034
14	0.779	0.283	<0.001	<0.001	0.507	0.009	0.001	0.083	0.034	0.424
15	0.001	0.108	0.002	0.083	0.507	0.14	0.283	0.424	0.596	0.596
16	0.596	0.424	0.507	0.859	0.859	0.968	0.859	0.779	0.779	0.968
17	0.924	0.689	0.003	0.034	0.424	0.002	0.034	0.006	0.002	0.046
18	0.349	0.001	<0.001	<0.001	0.108	<0.001	0.034	0.034	0.507	0.227

*One hundred nineteen out of 180 comparisons (61%) of the 5 within-rater trials were not significantly different (p >0.05). In these cases, raters made judgments of imitativeness consistently across rating blocks. The remaining 39% of the comparisons (shaded in gray), however, showed significant differences across ratings [χ^2^(9) = 143.32, p < 0.001], suggesting raters changed their decision patterns for ratings of imitativeness across the five trial blocks*.

There were 119 out of a total of 180 comparisons with non-significant differences (p-value <0.05). In other words, 61% of the comparisons show raters were overall consistent in their judgments, whereas the remaining 39% suggest raters changed their decision patterns across trials. A 2 × 2 chi-square test of independence determined that this pattern of listener changes across trials occurred at a rate much greater rate than chance, [χ^2^(9) = 143.32, *p* < 0.001].

## Discussion

The primary finding based on these data is that listeners were consistent both within their own repeated judgments and with other listeners on ratings of the degree of imitativeness in infant vocalizations from 3 to 12 months. Judgments of utterances inclusive of a wide range of imitativeness and lack of it evidenced moderate to strong relationships within and across raters, and these differences were highly significant statistically. The raters actually judged very few utterances as highly imitative—despite 48 out of the 166 pairs having been initially selected as being “highly imitative”—with only 5% of the mean ratings for the 166 pairs of parent and infant utterance exceeding 80 on the 100-point scale. Yet, the significant moderate to strong correlations indicate salience to the listeners of the imitative signal, even though it appears to have been weak. These results lead us to speculate that vocal imitation in the first year of life is a trait that may have undergone positive selection pressure as a fitness signal to indicate communicative well-being of the human infant.

The importance of the reliability of infant imitation as a signal seems augmented by the fact that imitation was observed rarely across the 36 recordings from which the stimulus materials were drawn. We found only 299 instances of utterance pairs where any degree of imitativeness was perceived by the stimulus selector out of 6,474 total infant utterances. These results are consistent with previous findings reporting that infant vocal imitation in naturalistic interactions does not occur frequently (Pawlby, 1977; Papoušek and Papoušek, 1989; Užgiris et al., 1989).

All in all, the results support an interpretation of the perception of infant vocal imitation that emphasizes salience of the imitation signal, as indicated by highly significant correlations among and within raters on judgments of utterances with regard to imitativeness. This salience suggests vocal imitation, though infrequent in occurrence, may serve as a fitness signal with regard to infant communicative abilities.

At the same time, the perception of the imitative signal shows variation in salience across different listeners as well as changes across time in judgments made within individual listeners. Trait variation among conspecifics is a primary postulate of Darwin's theory of evolution by natural selection (Darwin, 1859; Latta, 2010). The interpretation invokes the two evolutionarily necessary sides of imitativeness as an evolving trait: on the one hand it must show a measure of stability—reflected in fairly consistent perceptions of it—while on the other hand there must be variability in its perception, because without that, there would be no potential for natural selection of imitation as a fitness-signaling trait.

### Limitations and Future Directions

A limitation of this work relates to the small number of listeners as well as the selection of them, all being female, living in the USA. Also, all of them were associated with the IVOC laboratory with some experience identifying categories of infant sounds, but importantly with *no* experience rating degrees of imitativeness prior to participating in the study. Thus, our results cannot be generalized to all possible human listeners.

We have little reason to think the laboratory training that had been involved, namely training in infant vocalizations coding, had notable influence in our study. Four of the 18 listeners had engaged in some coding that had required them to label infant utterances for illocutionary force (Austin, 1962; Oller et al., 2016) where one of the possible categories was “imitation.” But, these four raters showed correlations very much like those of the other listeners and showed correlations with each other that were typical of the group. Even the first author, who was one of those four, and who had selected the stimuli, showed a typical agreement level with the others. Another important potential expansion of this work would be to compare male and female listeners. There have been other cases where gender differences have been found in perceptions of child development (Siegal, 1987; Kerig et al., 1993; Hastings et al., 2005), and consequently we cannot be sure that the patterns found here would apply equally to fathers or other male caregivers.

Future studies will hopefully assess listener differences by comparing experienced infant caregivers (individuals who have presumably made many tacit or explicit judgments about the imitativeness of infant vocalizations) with individuals having had little or no such experience. The two parents among the 18 listeners showed average rating agreement with the other listeners that was very near the mean for all the listeners, but because there were only two, we think further inquiry into a possible role for parenting experience is warranted. The experience of growing up in different cultures could also play a role, and we deem it important to evaluate judgments made by persons from different language and cultural backgrounds and presumably conditions of SES. Though we know of no research on vocal imitation rates being influenced by SES, there is a substantial literature on other kinds of SES effects in child development (e.g., Hoff-Ginsberg, 1998; Hoff, 2003; Conger and Donnellan, 2007).

Future directions for this line of research might also assess individual differences in rates of vocal imitation by infants. The current sample is too small (only six infants) to yield a persuasive picture on the matter, although the range of imitated utterances across the six infants was notable, from ≈ 22 per hour to ≈ 1 per hour (mean ≈ 10 per hour) in this sample of 6 recordings from each infant (see Appendix A). Similarly, the sample was too small to make much of gender differences, but the three girls had much higher rates (mean ≈ 16 per hour) than the three boys (mean ≈ 3 per hour). Although we know of no research on vocal imitation rates in naturalistic samples for boys and girls, there is of course a considerable literature base on gender differences in other realms of language development (e.g., Huttenlocher et al., 1991; Gleason and Ely, 2002). Furthermore, the girls tended to have mothers with higher educational levels—a common indicator of SES—than the boys. Another issue is that all the girls were first-borns whereas only one of the boys was (and he had the highest imitation rate among the boys ≈ 8 per hour). Again, we know of no research on imitation rates being affected by birth order, but there is a substantial literature on birth order effects other realms of child development (e.g., Breland, 1974; Zajonc and Markus, 1975;Hoff-Ginsberg, 1998).

Whatever the individual caregiving experience, gender, cultural or SES effects are determined in the future to be across a broad range of infants, it would also be useful to assess parents' perceptions of their *own* infant's imitation skills. Parents in our laboratory have sometimes asserted that their infants imitate frequently, suggesting that imitation is a salient indicator of vocal development for those individuals. It would be useful to determine whether parental perceptions correspond to the actual infant rates of imitation or whether either the rates or the parental perceptions of them are predictive of later vocal development.

## Ethics Statement

This study was carried out in accordance with the recommendations of the University of Memphis Institutional Review Board guidelines (https://www.memphis.edu/rsp/compliance/irb_forms.php) with written informed consent from all parents of infants in the study in accordance with the Declaration of Helsinki. The protocol was approved by the University of Memphis Institutional Review Board committee.

## Author Contributions

HL contributed to the conception, design, coding and statistical analyses, interpretation of results, and drafting/revising of written work. DO contributed to the conception, design, data collection, statistical analyses, interpretation of results, and revising of written work. DB contributed to the statistical analysis and interpretation of statistical results. All authors reviewed and approved final written draft (first submission to Frontiers).

### Conflict of Interest Statement

The authors declare that the research was conducted in the absence of any commercial or financial relationships that could be construed as a potential conflict of interest.

## Footnotes

^1^A second observer, blind to the purposes of the study, coded 11 recordings (30%), which had been selected at random from among the 36. A correlation of 0.88 was found across the 11 recordings between the primary and secondary observer on number of imitative utterances designated. The outcome for both coders on the selected recordings conformed to the widely reported tendency for vocal imitation to be found to occur rarely in infancy (see citations above), and in fact the second observer coded less than 2/3 as many items as imitative (16) as the primary coder (25) across the 11 recordings.

^2^Four raters (Raters 1, 2, 10, and 11) had previous coding experience identifying illocutionary forces of infant utterances, including a category labeled *Imitation*. However, training for this category included only the brief presentation of a list of auditory-perceptual criteria to consider for imitation. The raters were instructed to make their judgments based on intuition. Rater 1, who selected the stimuli, and worked closely with the last author, was the only member of the group that could be thought to have engaged in a sort of training on imitation.

^3^One listener reported selecting a “Show Rating” option that was available on the rating scale, which resulted in a display of the digital value (0–100) associated with the position on the visual scale for each placement of the cursor. The remaining listeners did not see the numerical values.

## Supplementary Material

**Data Sheet 1**, plus ten Audio waves, captions for which can be found in Appendix D of **Data Sheet 1**.
