# Supplementary material for: Reliability of Listener Judgments of Infant Vocal Imitation
Source: Front Psychol. 2019 Jun 11;10:1340. doi: 10.3389/fpsyg.2019.01340 (PMC6579846; doi:10.3389/fpsyg.2019.01340)
Supplement: Supplementary file 1 [file Data_Sheet_1.pdf]

Supplementary Material

Appendix A: Infants

A1. Infant demographics

Infant	Gender	Birth order	Maternal education	Ethnicity	Home language
1	Female	1	PhD	White/Caucasian	English
2	Female	1	Some graduate school	White/Caucasian	English
3	Female	1	PhD	White/Caucasian	English, Ukrainian
4	Male	3	Some college	White/Caucasian	English
5	Male	2	BA	White/Caucasian	English
6	Male	3	Some college	White/Caucasian	English

Table A1. Infant demographics.

A2. Infant ages in recordings used for stimulus selection

Infant	Gender	Recording age of infant			Imitations per minute
1	F	3 mo 1 wk 4 dy	6 mo 0 wk 6 dy	9 mo 4 wk 1 dy	0.36
		3 mo 1 wk 4 dy	6 mo 3 wk 3 dy	9 mo 4 wk 1 dy	
2	F	4 mo 0 wk 2 dy	6 mo 0 wk 3 dy	11 mo 3 wk 2 dy	0.22
		4 mo 1 wk 2 dy	7 mo 1 wk 0 dy	11 mo 3 wk 2 dy	
3	F	3 mo 0 wk 4 dy	5 mo 0 wk 4 dy	10 mo 1wk 6 dy	0.25
		3 mo 0 wk 4 dy	6 mo 0 wk 4 dy	10 mo 1 wk 6 dy	
4	M	3 mo 2 wk 5 dy	6 mo 0 wk 3 dy	9 mo 3 wk 6 dy	0.02
		3 mo 2 wk 6 dy	6 mo 3 wk 6 dy	9 mo 3 wk 6 dy	
5	M	4 mo 2 wk 2 dy	6 mo 0 wk 4 dy	11 mo 2 wk 1 dy	0.02
		4 mo 2 wk 2 dy	7 mo 3 wk 1 dy	11 mo 2 wk 1 dy	
6	M	3 mo 2 wk 0 dy	5 mo 0 wk 2 dy	10 mo 0 wk 6 dy	0.13
		3 mo 2 wk 0 dy	6 mo 0 wk 2 dy	10mo 0 wk 6 dy	
**Average**		**3 mo 2 wk 3 dy**	**6 mo 1 wk 3 dy**	**10 mo 2 wk 4 dy**	**0.16**

Table A2. Infant ages in recordings used for stimulus selection. Imitations per minute offers perspective on possible individual differences in rate of imitation.

Appendix B: Stimulus pair selection

B1. Visualization of selection process for stimulus pairs

A number of labels were used heuristically during stimulus selection, but the experiment did not utilize these category labels to designate any aspect of imitativeness. Instead the study with the 18 listeners addressed a continuum of imitativeness only. There was only a preliminary attempt to match the number of selected items in the no, low, and high imitation groups, and we did not view such matching as important since the focus was a single continuum rather than categories.

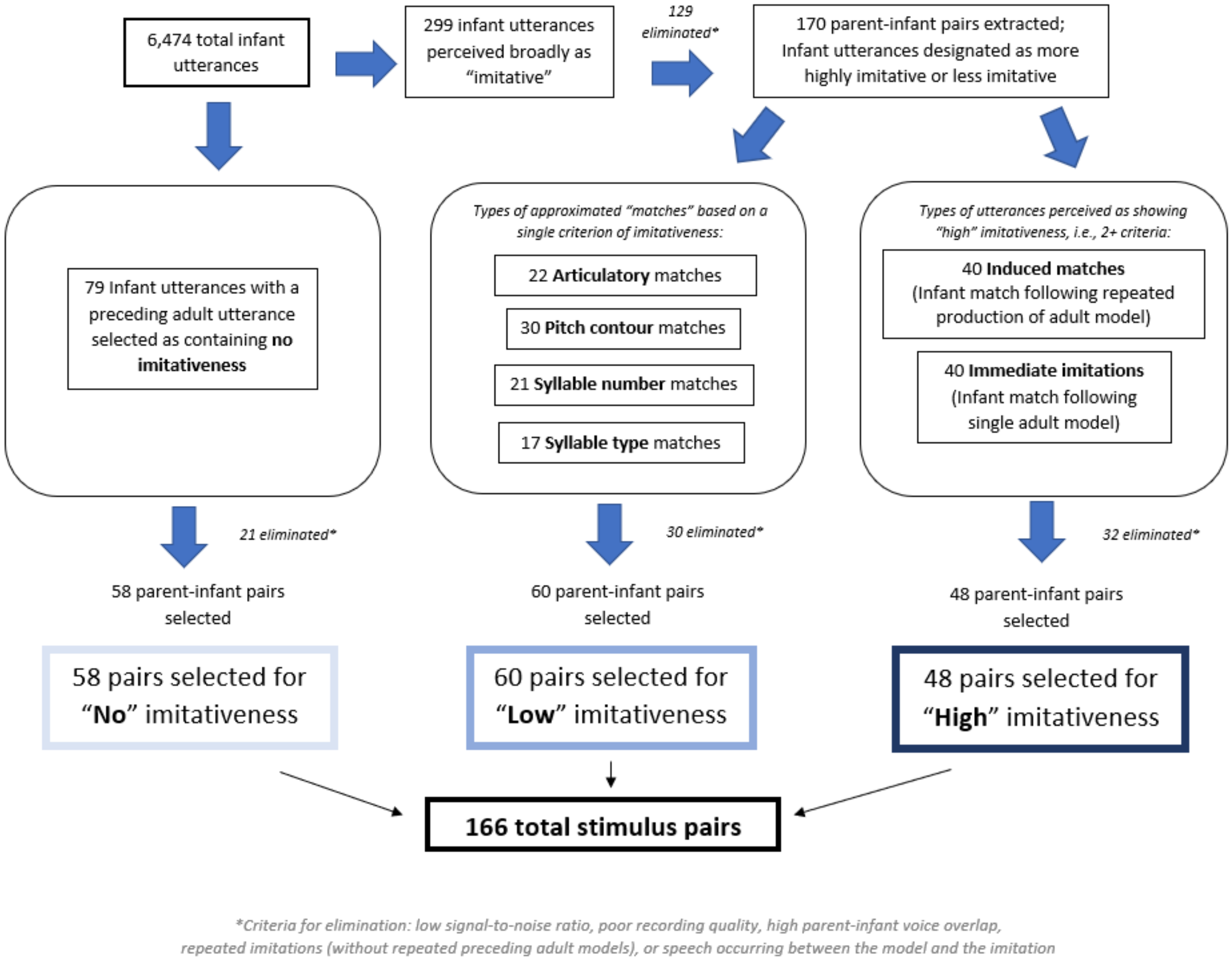

Figure B1. Visualization of stimulus selection process

Appendix C: Rating scale

C1. Rating scale

Picture C1 provides a screen shot of the continuous rating scale presented to listeners for making judgments on the degree of infant imitation. Listeners selected “Play” to hear a stimulus pair, then selected a position somewhere along the scale to rate *how* imitative the infant vocalization was compared to the adult model. Listeners pressed “Next” to continue and completed the task after 830 total ratings (166 stimulus pairs, 5 randomized blocks).

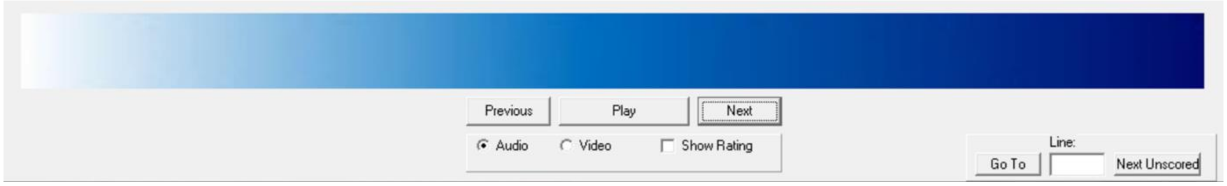

Picture C1. Continuous rating scale presented to listeners for making judgments on the degree of infant imitation.

C2. Rating scale usage and variation

To estimate the variability in individual stimulus pair ratings, we computed the mean rating (individual rater means, IRMs) across the 5 trials on each stimulus pair for each listener. We then calculated the stimulus pair means (SPMs) for ratings of each stimulus pair, that is, the means of the IRMs across the 18 raters. We similarly calculated the stimulus pair standard deviations (SPSDs). *Figure C2* presents the SPMs versus the SPSDs, thus characterizing the consistency across trial judgments for each of the 166 pairs, aggregating the ratings from all 18 listeners. The parabolic shape of the distribution suggests that listeners were consistent in their judgments of very low and very high degrees of imitativeness but had greater variability in rating items for moderate levels of imitativeness. In other words, the consistency of judgments was not uniform across the range of trials and was greater for extreme judgments of "not imitative" and "highly imitative."

Ratings for the 12 calibration stimulus pairs are represented as red and blue triangles—low and high imitativeness, respectively—in *Figure C2*. These pairs had been selected by the first author and explicitly presented to listeners prior to the judgment task as examples of very low and very high degrees of imitativeness. The listeners consistently rated the low calibration pairs as having a low degree of imitativeness (M = 8.78, SD = 7.08), whereas the high calibration pairs were rated with greater variability (M = 74.12, SD = 11.42). Rater 1 rated all the low calibration pairs $\leq$ 5, and all the high calibration pairs $\geq$ 80.

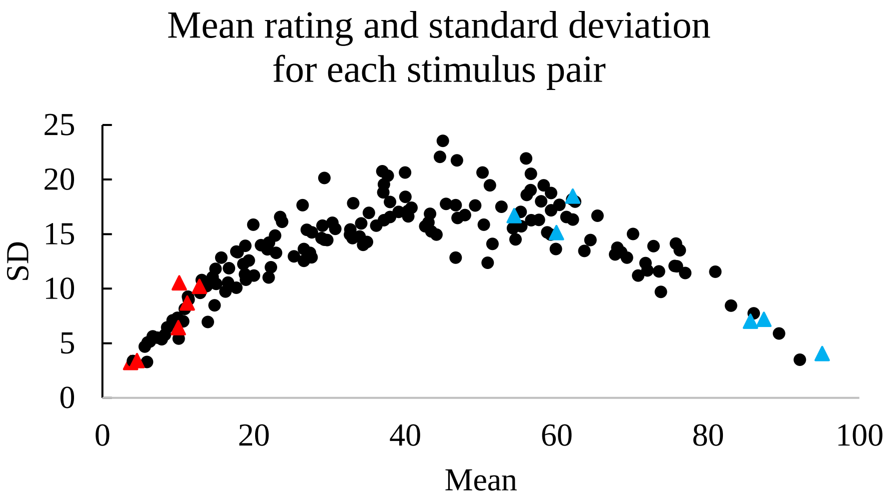

Figure C2. Dots and triangles represent the average of the means and standard deviations across all 18 raters on each individual stimulus pair (N = 166). The data show listeners used more consistent ratings for extremely low and high degrees of imitativeness. ● = Stimulus pair not among the calibration items; ▲ = Low imitativeness calibration item, ▲= High imitativeness calibration item.

C3. Frequency distribution of rating scale usage

An analysis of overall rating bias was calculated on the frequencies of individual rating values across the 0-100 scale as seen in *Figure C3* (grouping 90-100 included 11 values; all other groupings included 10 values, i.e. there were 101 possible rating values in the scale from 0-100). With 18 raters and 5 stimulus-pair trial blocks of 166 items, there were a total of 14,940 ratings for the entire experiment. Lower rating judgments were used more often, suggesting a tendency to judge the infant utterances as having a low degree of imitativeness. Specifically, the total number of ratings from 0 to 9 made up 29.0% of the total of all the ratings, whereas each of the other rating intervals made up on average 7.9% of the total.

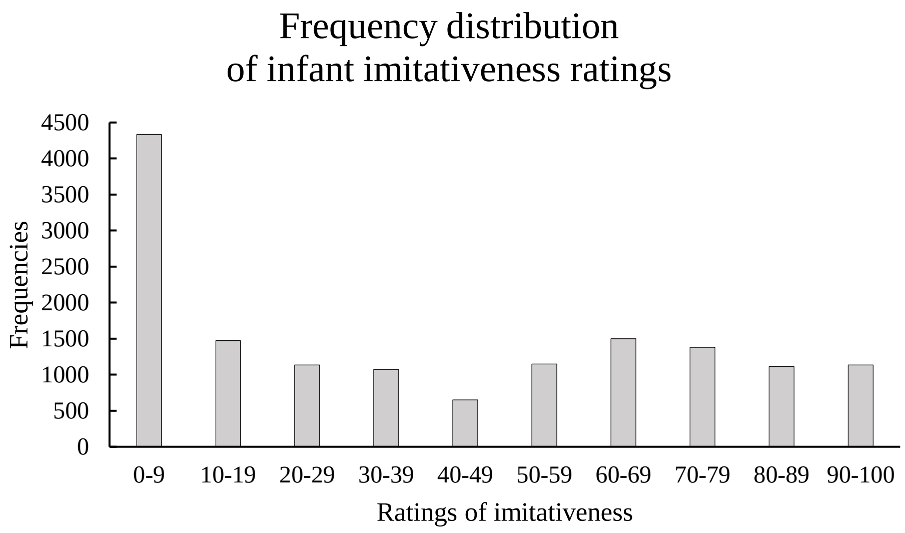

Figure C3. Frequency distribution of the 14,940 ratings (166 stimuli x 18 listeners x 5 trials) used across the 0-100 scale. Listeners predominantly rated utterances as having a low degree of imitativeness (0-9).

C4. Display of mean individual rater bias (an intra-rater analysis)

Mean ratings of each listener across the five trial blocks were calculated to examine individual biases regarding degree of rated imitativeness, as displayed in *Figure C4*. The average rating of individual listeners was 39.3 (range: 16.2-55.2). Listeners consistently rated pairs as having a relatively low degree of imitativeness; all but three raters had an average rating below 50. The figure shows that the listeners significantly differed in rating bias (or criterion). These differences are reflected in the means and 95% CIs. Note in particular Rater 11, who shifted from a first trial mean rating of 17.9 to a fifth trial mean of 45.6. This suggests she changed her criterion or rating bias substantially across the trials. On the other hand, Raters 8, 9, 12, 16, and 2 scarcely changed their rating criteria across the five trials.

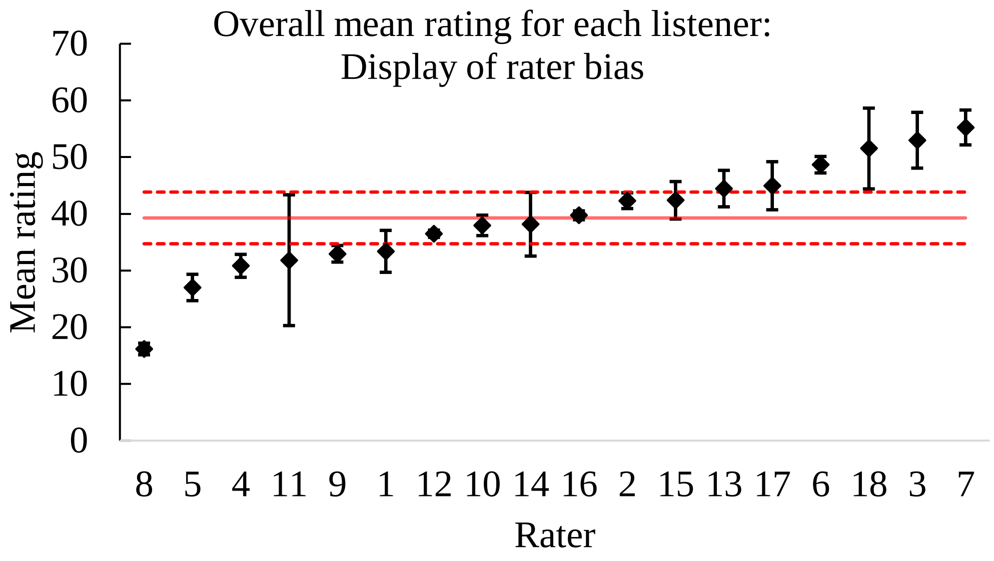

Figure C4. Mean ratings for each listener, ordered from lowest (M = 16.2) to highest (M = 55.2), with 95% confidence intervals represented for each. Y-axis reflects range of rating scale, 0-100. The overall mean rating was 39.3 (95% CI = 34.7 – 43.8).

C5. Rating bias across stimuli between raters (an inter-rater analysis)

Evaluating rater bias differences between listeners, we compared each rater with all others on their mean ratings across the 166 pairs. Paired t-tests were calculated to compare IRMs across the 18 raters. Specifically, the IRM for each rater (N = 18) was compared to the IRMs for all other raters, yielding a total of 153 possible paired comparisons t-tests (n=166) as seen in the Figure below. 130 out of the 153 comparisons were found to be significantly different ($p < .05$), suggesting raters were making judgments the means of which were systematically different from those of other raters, that is, that the raters showed different rating biases. In other words, 85% of the comparisons showed strong differences in ratings between listeners. A 2x2 chi-square test of independence supports the idea that listeners were systematically different from each other in their perceptions of the degree of imitativeness in stimulus pairs, $\chi^2(17) = 101.69$, $p < .001$. It is important to emphasize, however, that the bias differences between raters are independent of the correlations that obtained among raters. Even though the bias differences were very discernible and statistically significant, it is also true that the raters showed strong agreement in terms of correlations of their ratings with each other.

Paired t-test comparisons of rater IRMs across individual stimulus pairs.

	2	3	4	5	6	7	8	9	10	11	12	13	14	15	16	17	18
**1**	<.001	<.001	0.096	<.001	<.001	<.001	<.001	0.778	0.030	0.358	0.058	<.001	0.005	<.001	0.001	<.001	<.001
**2**		<.001	<.001	<.001	<.001	<.001	<.001	<.001	0.036	<.001	<.001	0.209	0.017	0.962	0.158	0.099	<.001
**3**			<.001	<.001	0.019	0.095	<.001	<.001	<.001	<.001	<.001	<.001	<.001	<.001	<.001	<.001	0.412
**4**				0.016	<.001	<.001	<.001	0.180	0.001	0.559	0.001	<.001	<.001	<.001	<.001	<.001	<.001
**5**					<.001	<.001	<.001	0.001	<.001	0.012	<.001	<.001	<.001	<.001	<.001	<.001	<.001
**6**						<.001	<.001	<.001	<.001	<.001	<.001	0.010	<.001	<.001	<.001	0.012	0.078
**7**							<.001	<.001	<.001	<.001	<.001	<.001	<.001	<.001	<.001	<.001	0.015
**8**								<.001	<.001	<.001	<.001	<.001	<.001	<.001	<.001	<.001	<.001
**9**									0.006	0.452	0.008	<.001	0.001	<.001	<.001	<.001	<.001
**10**										0.001	0.391	<.001	0.917	0.010	0.210	<.001	<.001
**11**											0.002	<.001	<.001	<.001	<.001	<.001	<.001
**12**												<.001	0.297	<.001	0.026	<.001	<.001
**13**													<.001	0.160	0.001	0.696	<.001
**14**														0.012	0.279	<.001	<.001
**15**															0.095	0.080	<.001
**16**																0.001	<.001
**17**																	<.001

Table C5. 130 out of 153 comparisons (85%) were found to be significantly different ($p < .05$), suggesting raters were making judgments that were systematically different from each other in terms of bias. Thus Rater 1's mean judgments on the 166 stimuli were statistically different from those of Raters 2, 3, 5-8, 10, and 13-18 (either higher or lower in each case).

Appendix D: Audio wave files

D1. Audio wave file mean and standard deviations

File	Mean Rating	SD
Audio 1.WAV	3.90	3.40
Audio 2.WAV	6.13	3.40
Audio 3.WAV	25.01	13.36
Audio 4.WAV	23.13	13.04
Audio 5.WAV	50.34	14.41
Audio 6.WAV	51.57	18.21
Audio 7.WAV	75.39	12.03
Audio 8.WAV	72.13	11.73
Audio 9.WAV	93.40	6.31
Audio 10.WAV	86.25	8.3

Table D1. Audio wave file information. Raw rating mean and SD of individual audio files across all raters and judgments.
